# Effects of High Temperature on the Burst Process of Carbon Fiber/PVA Fiber High-Strength Concretes

**DOI:** 10.3390/ma12060973

**Published:** 2019-03-24

**Authors:** Rui-dong Cao, Hui-wei Yang, Guo-yun Lu

**Affiliations:** 1 Shanxi Key Lab of Material Strength & Structural Impact, Taiyuan University of Technology, 79 Yingze Street, Taiyuan 030024, China; caoruidongcrd@126.com; 2 College of Architecture and Civil Engineering, Taiyuan University of Technology, 79 Yingze Street, Taiyuan 030024, China; hebyanghuiwei@163.com

**Keywords:** high strength concretes, burst process, carbon fiber, PVA fiber

## Abstract

This paper carried out burst tests on the carbon and polyvinyl alcohol (PVA) fiber high-strength concrete specimens to investigate the effects of fiber type, fiber content, water content, heating rate and test specimen size on the burst, and the whole burst process of fiber-high concrete was photographed and recorded. The results indicated that fiber addition will improve the high temperature burst behavior of the high-strength concrete, and the performance of PVA is greatly different from that of carbon fiber. The water content and heating rate have little influence on the burst of the PVA test specimen, but they will greatly affect the carbon fiber test specimen. The size of the test specimen has a great influence on the burst. For the PVA concrete test specimen, the large size test specimen bursts on the surface; as for the carbon fiber test specimen, the large size test specimen delays the initial burst time, but the burst becomes fiercer.

## 1. Introduction

High-strength concrete possesses superb mechanical properties [1,2,3,4,5] and durability, which has been extensively applied in practical engineering [6,7]; however, it is likely to burst under high temperatures [8,9]. Some studies indicate that high-strength concrete is subject to bursting at the temperatures of 300–500 °C [10,11,12,13,14,15], and scholars have proposed three theories, namely, vapor pressure theory [16,17,18], thermal stress theory [19] and temperature gradient theory [20,21], to explain the cause of bursting. Moreover, it has been proposed that the addition of fiber in high-strength concrete can alleviate the occurrence of bursts. Typically, the burst-proof fibers can be classified into two categories based on the basic theory, namely, the high elastic modulus fiber dominated by steel fiber, which can be ascribed to the reinforcement, tensile strength and high thermal conductivity of steel fiber; the other type of fiber is the plastic fiber dominated by Polypropylene (PP) fiber, which can form the pore canal under high temperatures and can release the vapor pressure to alleviate the occurrence of bursts. Meanwhile, scholars have investigated the influence of fiber content; for instance, Yuh-Shiou Tai [22] and Liu Hongbin [23] pointed out that 2% steel fiber could alleviate the burst of high-strength concrete at high temperatures. Scholar [24] studied the influence of PP fiber on the burst of high-strength concrete and discovered that a 0.2% volume content of PP fiber could avoid the occurrence of bursts, whereas ultrahigh-strength concrete with a 0.3% PP fiber content would burst under high temperatures, indicating the presence of a suitable zone in the plastic fiber content. 

As a matter of fact, there are fibers with superior properties other than steel fiber and PP fiber, which can be used to improve the property of high-strength concrete; for instance, carbon fiber and polyvinyl alcohol (PVA) fiber have better mechanical properties than steel fiber and PP fiber. The tensile strength of carbon fiber can be as high as 4200 MPa; moreover, it has excellent thermal conductivity, but its diameter and length are far lower than those of steel fiber. In addition, PVA also has superior mechanical properties to PP fiber but with a lower diameter, which also belongs to the low-melting-point plastic fiber. However, whether the addition of these two fibers into the high-strength concrete can exert a similar burst suppression effect to that of steel fiber and PP fiber remains unclear. It was only pointed out in the research by Han [25] that a 0.1% PVA content could prevent bursts, but whether excessive fiber could exert a burst suppression effect similar to that of PP fiber has rarely been studied in domestic and foreign studies, while the high temperature burst process of PVA and carbon fiber high-strength concrete has never been reviewed in the literature. It is of crucial necessity to determine these problems to understand the burst mechanism of high-strength concrete and to provide a new thinking for the high temperature burst suppression of high-strength concrete. Therefore, this paper aimed to examine the burst process of carbon fiber/PVA fiber high-strength concrete from the aspects of fiber type, fiber content, water content, heating rate and specimen size.

## 2. Experiment 

### 2.1. Experimental Materials and Mixture Ratio

The major materials of the concrete were as follows: The Ordinary Portland cement (Taiyuan Shitou Cement Co. Ltd, Taiyuan, Shanxi, China) had minimal compressive and rupture strengths of 42.5 MPa and 6.5 MPa, respectively; the specific surface area of the mineral powder was 4750 cm^2^/g; the specific surface area of the silica fume was 207800 cm^2^/g, the ignition loss quantity was 0.31%; the specific surface area of the ultrafine fly ash was 10600 cm^2^/g, the ignition loss quantity was 7.2%; the fineness modulus of the river sands was 2.45; The scope of the diabase’particle size was 5–10 mm; the crushing index was 4.6%; the polycarboxylate super plasticizer had a water-reducing rate of 30%; and the dry matter content was 50%. By adjusting the amount of water reducing agent, the slump of concrete was maintained at 200–220 mm. The mixture ratio is presented in Table 1. C2 represented the carbon fiber content, which was 0.2% of the concrete volume content, while P2 suggested that the PVA fiber content was 0.2% of the concrete volume content. The property parameters of carbon fiber and PVA fiber are presented in Table 2. 

### 2.2. Molding Techniques

The materials were precisely weighed according to the mixture ratio in Table 1; firstly, the powder and fiber were stirred for 120 s, followed by the addition of additives and water stirred for 240 s, the addition of sands stirred for 60 s and the addition of aggregates sufficiently stirred. Then, the mixture was put in the mould and vibrated in the high-frequency vibrating tamper (Wuxi Jianyi Instrument & Machinery Co. Ltd, Wuxi, Jiangsu, China) for 30 s, followed by standard curing for 28 days. After 28 days of standard curing, the test block was placed outside for a period of time for the test. The following mechanical experiments at room temperature were carried out: compression resistance, axial compression, flexural strength and splitting tests of the concrete. Three specimens were made for each mechanical property, and the average values of the three specimens were taken. According to the Standards of Methods for Mechanical Property Test of Ordinary Concrete (in China), the dimension of 100 mm × 100 mm × 100 mm was used for the test specimens for the compression resistance and splitting tests, while 100 mm × 100 mm × 300 mm was adopted for the axial compression test specimens and 100 mm × 100 mm × 400 mm was used for the flexural strength test specimens; in addition, two dimensions of 100mm × 100 mm × 100 mm/150 mm × 150 mm ×150 mm were used for the burst test specimens. 

### 2.3. Experimental Apparatus

**Figure 1:**
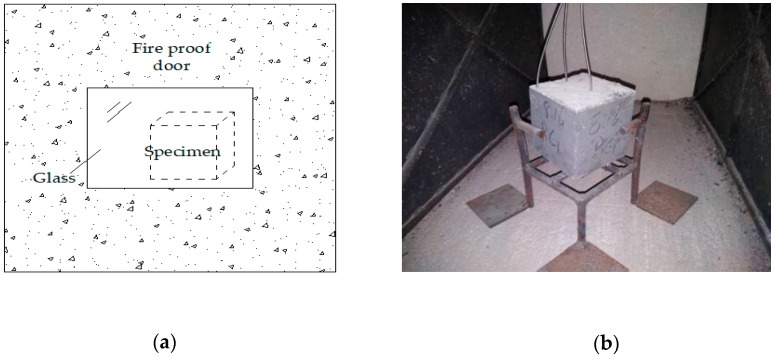
The test equipment: (**a**) the self-made fire-proof door and (**b**) steel frame.

The high-temperature test furnace adopted for heating the concrete was a self-processed electric furnace high-temperature chamber furnace (Luoyang Zhongyuan Experimental Furnace Factory, Luoyang, Henan, China). The rated power was 18 kW, the maximum operating temperature was 1200 °C, and the hearth size was 800 mm × 500 mm × 500 mm; in addition, it was equipped with an automatic temperature control box (Luoyang Zhongyuan Experimental Furnace Factory, Luoyang, Henan, China), which could set the target temperature and heating rate and could automatically change to a constant temperature after reaching the target temperature. The furnace door was made of aluminate cement and had set up the refractory glass (Figure 1a), which allowed for photo taking of the failure process of the high-strength concrete. The electric furnace was dark in the interior, and a highlight light source was set externally so that the internal electric furnace could be lit up and clear pictures could be taken to visualize the changing status of the test specimen in a real-time manner. To guarantee that the test specimen could be evenly heated in the electric furnace, the concrete should be risen using the self-made steel frame, as displayed in Figure 1b. 

### 2.4. Test Method

Previous studies had indicated that the fiber type and content [26,27], the water content [28,29], the heating rate [29,30] and the specimen size [30] had great influences on the burst process of the high-strength concrete. This paper first tested the mechanical properties of the concrete test specimens at various mixture ratios at room temperature and then explored the burst process of carbon fiber and PVA fiber high-strength concrete from the above five aspects. The water content was divided into drying and natural moisture content; of them, drying was performed using the 90 °C drying box (Beijing Zhongxing Weiye Instrument Co. Ltd, Beijing, China) under ongoing drying until the 2 h weight difference of the test specimen was less than 2 g [31], which was considered to have 0% water content; the natural water content referred to the water content after being placed outdoors for a long time. There were two test specimen sizes, namely, 100 mm and 150 mm cubes, and the heating rates were 3 °C/min and 5 °C/min, respectively, Due to instrumental reasons, there is a little deviations in heating temperature. The precise test conditions for each research content are shown in Table 3. 

## 3. Mechanical Property at Room Temperature and Working Performance

Ordinary concrete has an excellent fire-resistance performance, which will not burst under high temperatures. Denmark scholar Hertz [32] had first proposed that high-strength concrete might easily burst under high temperature. Thereafter, numerous studies have also verified that high strength concrete would easily burst at high temperatures, and the risk of incidences of bursting is increased with the increase in strength. Therefore, the burst degree is closely correlated with the concrete strength at room temperature; as a result, the room temperature mechanical property and working performance of the test specimens should be tested before a burst test (Table 4 and Table 5). The fresh concrete has a good water retention and flow ability.

### 3.1. Compression Strength

**Figure 2:**
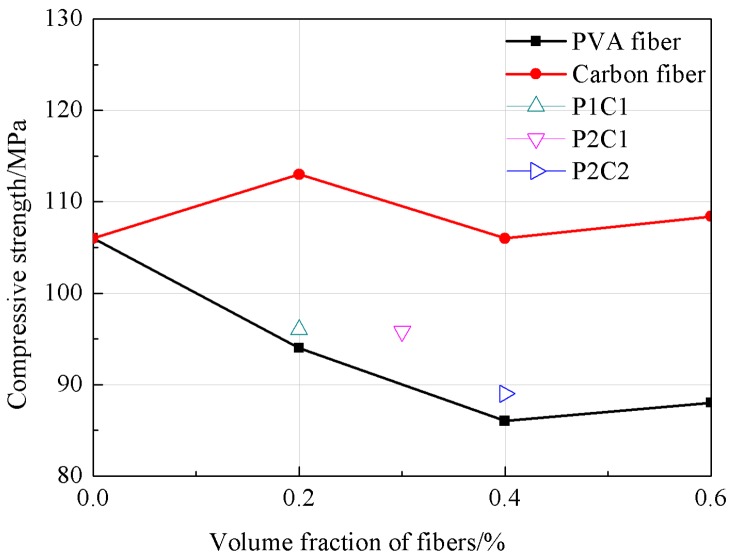
The tendency of the compressive strength.

As could be seen from Figure 2, the compression strength of PVA fiber concrete was rapidly decreased at the fiber content of 0.2%, while that of carbon fiber concrete was elevated. Compared with the reference concrete, the compression strength of PVA fiber concrete was reduced by 11%, while that of carbon fiber concrete was elevated by 6.6%; moreover, at a fiber content of 0.6%, that of PVA fiber concrete was reduced by 17% compared with the reference concrete, while that of carbon fiber concrete was increased by 2% relative to the reference concrete. Thus, it could be found that the compression strength of the high strength concrete was decreased with the increase in the PVA fiber volume content and increased with the carbon fiber volume content. The compression strength of specimen of P + C was between PVA fiber concrete and carbon fiber concrete.

### 3.2. Splitting Strength

**Figure 3:**
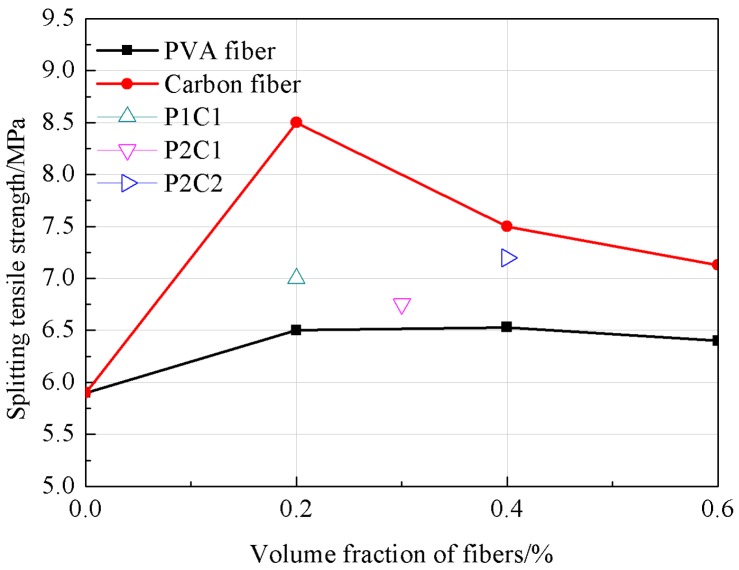
The tendency of the splitting tensile strength.

It could be observed from Figure 3 that the fiber content would improve the splitting tensile strength of the high-strength concrete to various degrees. When the fiber content was increased from 0.2% to 0.4%, the splitting tensile strength of PVA fiber concrete was basically unchanged; when the fiber content was 0.6%, the splitting tensile strength of PVA fiber concrete was increased by 8%. While the fiber content was increased from 0.2% to 0.6%, that of carbon fiber was increased by 44–20.3%. Thus, it could be found that, with the addition of PVA fiber and carbon fiber, the high elastic modulus fibers were irregularly arranged inside the concrete, which had formed a reticular system. In addition, there was an adhesion strength [33] between the fiber and concrete, which could suppress the development of the microfracture, improve the concrete toughness and thereby improve the splitting tensile strength of the concrete. 

### 3.3. Flexural Strength

**Figure 4:**
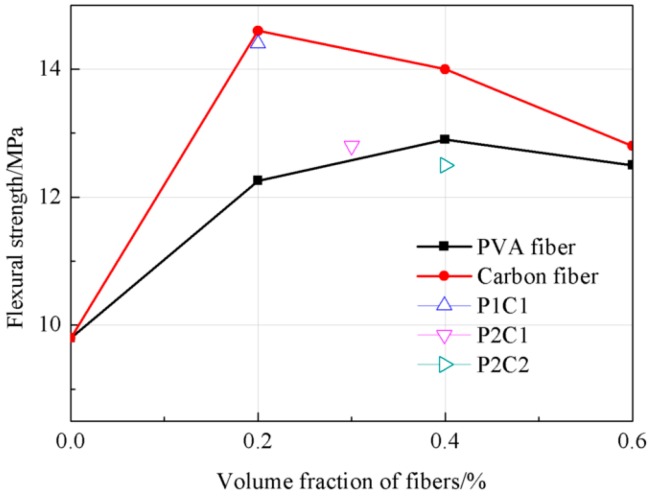
The tendency of the flexural strength.

As could be observed from Figure 4, the flexural strength of the high-strength concrete was increased with the increase in fiber volume content; the most remarkable increase in the flexural strength of the high-strength concrete could be achieved at the fiber content of 0–0.2%, which was increased by 25.5% in PVA fiber concrete and 49% in carbon fiber concrete. At a fiber content of 0.6%, the flexural strength of PVA fiber and carbon fiber concretes was elevated by 28% and 31%, respectively, relative to the reference concrete. During the experimental process, the reference concrete had manifested a typical brittle failure, while that was plastic failure in fiber concretes; the fiber concrete test specimens only manifested a failure in the test until the fibers were snapped or pulled out, which could be ascribed to the connection of fibers in the concretes. 

### 3.4. Axial Compression Strength

**Figure 5:**
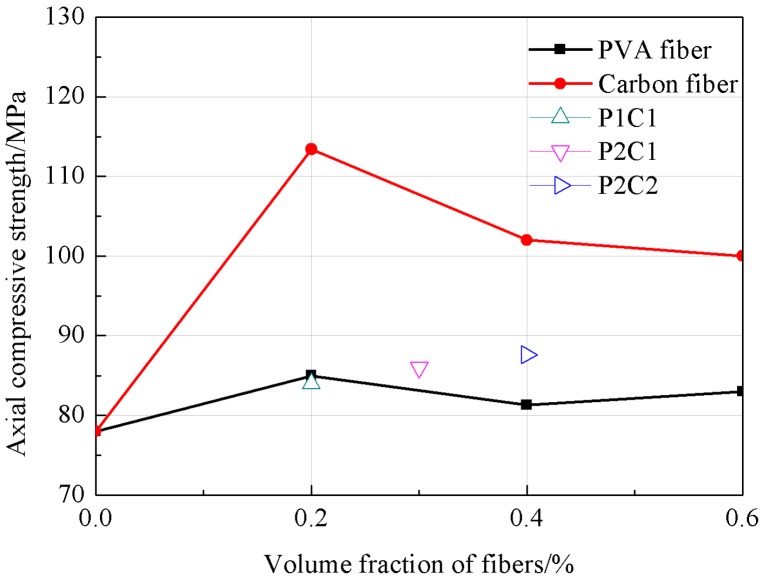
The tendency of the axial compressive strength.

It could be seen from Figure 5 that, with the increase in fiber volume content, the axial compression strength of the fiber concrete was slightly increased. At the fiber volume contents of 0.2%, 0.4% and 0.6%, the axial compression strength of the PVA fiber concrete was increased by 9%, 4% and 6 %, respectively, compared with the reference concrete, while that of carbon fiber concrete was increased by 45%, 31% and 28%, respectively, compared with the reference concrete. Based on the mechanical properties of fiber concrete, it can be found that the mechanical properties of the P + C specimen were between the carbon fiber specimens and PVA fiber specimens.

## 4. Burst Process of the Test Specimens

**Figure 6:**
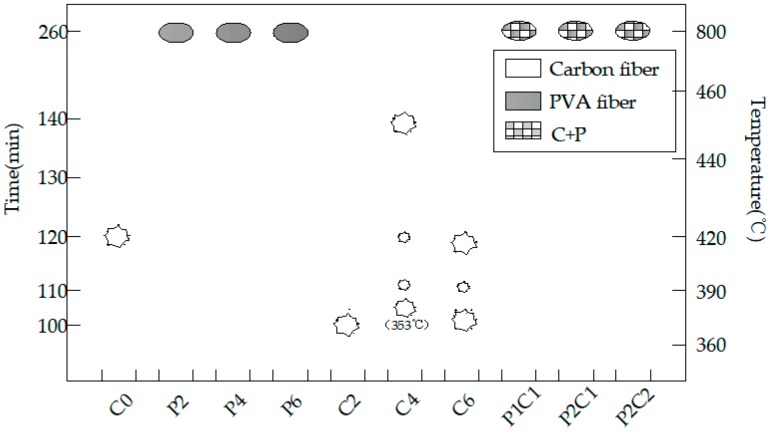
The burst statistics of concrete with different fiber contents.

Photos were taken at an interval of 30 min to record the change status of the high-strength concrete test specimens under high temperature, and photos of the change status of test specimens within the high-temperature furnace were taken at an interval of 10 min at the presented burst intensive time zone. At the same time, the burst sounds were also recorded. In the burst statistical charts (such as Figures 6, 9, 12 and 15), the greater polygons indicated greater bursts, while the smaller polygons suggested smaller bursts, and the ellipses indicated non-explosion.

### 4.1. Effect of Fiber Content

**Figure 7:**
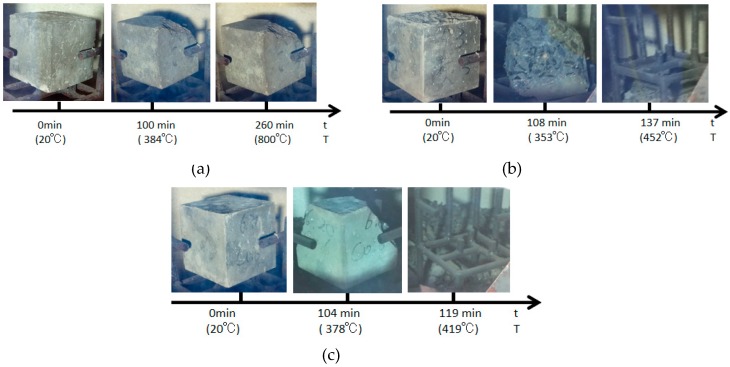
The burst process of the carbon fiber specimens with different dosages: (**a**) the burst process of C2, (**b**) the burst process of C4 and (**c**) the burst process of C6.

The burst test results are displayed in Figure 6, while Figure 7 presents the precise burst process of test specimens with different carbon fiber contents. It could be discovered from Figure 6 and Figure 7 that the test specimen without the addition of any fiber developed a fierce burst at 120 min into powder. The test specimen with a 0.2% carbon fiber content was the first to develop initial corner spalling at 100 min, accompanied by an explosive sound; at this time, the furnace temperature was 384 °C. By contrast, the remaining test specimens with 0.4% and 0.6% fiber contents began to burst within the subsequent 10 min. 

The test specimen with a 0.2% carbon fiber content had spalling once only, the upper part of this test specimen as well as half of the lateral part burst, but no burst occurred thereafter (Figure 7a). At the initial burst, the upper part of the entire test specimen with a 0.4% carbon fiber content had developed destructive spalling, the volume of which almost accounted for half of the entire specimen, and the furnace temperature at this time was 353 °C; the second burst occurred at 137 min: The test specimen had completely burst at this time, a “bang” sound was heard, the specimen had completely burst into pieces, no burst was seen thereafter and the furnace temperature at this time was 452 °C (Figure 7b). 

The initial burst of the test specimen with a 0.6% carbon fiber content occurred in the side back to the observational glass, the seamed edges and planes of the test specimen were exploded and the furnace temperature at this time was 378 °C; subsequently, a huge burst sound was heard at 119 min, the test specimen had completely burst, the burst fragments could be seen around and the furnace temperature at this time was 419 °C (Figure 7c). High-strength concrete and carbon fiber high-strength concrete will not burst when the temperature is lower than 300 °C. When the temperature is higher than 300 °C, it is very easy to burst.

**Figure 8:**
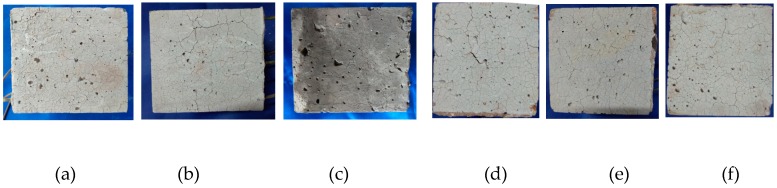
The final state of the P and P + C specimens: (**a**) P2, (**b**) P4, (**c**) P6, (**e**) P1C1, (**d**) P1C2 and (**e**) P2C1.

In this experiment, the addition of PVA fiber, regardless of whether the carbon fiber was mixed, did not burst, but there were numerous cracks on the surface, as presented in Figure 8. It could be discovered based on the test results that, for the 100-mm cubic test specimens, the addition of at least a 0.2% volume content of PVA fiber could prevent bursts, and the 0.1% PVA + 0.1% carbon fiber test specimen could also exert the explosion-proof effect in Figure 8. When the volume fraction of PVA fibers is more than 0.1%, there will hardly be any bursting.

It could be discovered based on the abovementioned experiments that, when compared with the initial burst time of the reference concrete, the presence of carbon fiber would advance the initial burst time of the concrete and that the addition of carbon fiber would change the sudden complete burst into a continuous and gradual burst. The addition of a small amount of carbon fiber would alleviate the burst, but the burst degree would be aggravated with the increase in carbon fiber content; typically, carbon fiber test specimens with 0.4% and above volume contents would finally completely burst. However, no great difference was observed in the initial burst time of the carbon fiber test specimens, which occurred at around 100 min, and the furnace temperature at this time was about 350 °C. By contrast, the PVA and C + P test specimens had not burst. 

The possible reason was that carbon fiber possessed an excellent tensile strength; with the increase in temperature, the concrete would remarkably expand under heat treatment, while the thermal expansion coefficient of carbon fiber was quite small, which finally manifested as almost unchanged. This, together with the steam pressure, would lead to the increase of the bond stress enven surface slip occure on the contact surface, and bursting would emerge when the stress was greater than the concrete tensile stress. The PVA melting point was 230 °C; with the increase in temperature, the heat would be transferred gradually from the outer surface to the interior, while PVA would be melted from outside in, and the site where PVA used to exist would be melted to form the small pores. On the other hand, the water in the concrete would absorb the heat to become steam, which would thereby fill these pores and be circulated; at the same time, the pores could also accumulate more energy to alleviate the steam pressure, thus avoiding the occurrence of burst. 

### 4.2. Effect of Water Content

**Figure 9:**
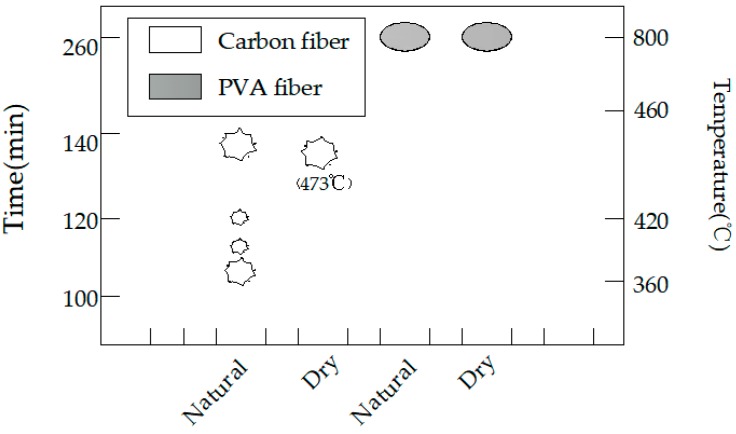
The burst statistics of concrete with different water contents.

**Figure 10:**
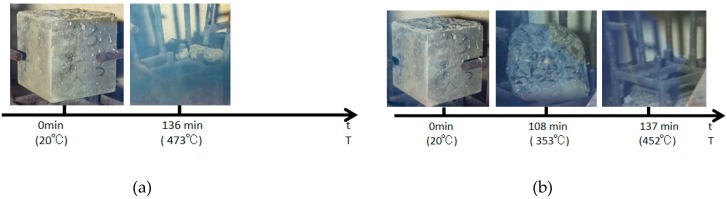
The explosion process of the carbon fiber specimens with different water contents: (**a**) Dry and (**b**) natural.

**Figure 11:**
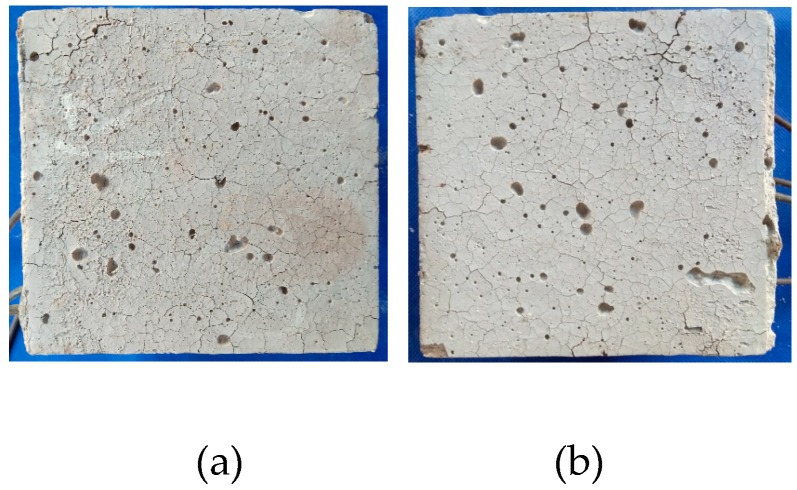
The final state of the PVA fiber specimens with different water contents: (**a**) Dry and (**b**) natural.

The burst statistics of the test specimens with various water contents are shown in Figure 9. The carbon fiber test specimens, regardless of a high or low water content, would burst, while the PVA test specimens did not burst. Moreover, it could be discovered from the burst process shown in Figure 10a that the C4 dried test specimen had only one fierce burst, and it completely burst at 136 min, with the furnace temperature of 473 °C at this time. For the C4 test specimen dried in the air, it burst twice when heating to 800 °C; the first one was at 108 min, the upper half of the test specimen exploded, which was nearly one half of the volume, and the furnace temperature at this time was 353 °C; the second burst occurred at 137 min, the test specimen had completely burst into fragments, and the furnace temperature at this time was 452 °C (Figure 10b). By contrast, PVA fiber concretes with a volume content of 0.4% did not burst, but dense cracks could be seen on the surface, as displayed in Figure 11. 

It could be observed when comparing the burst status of carbon fiber test specimens with two water contents that the test specimen with a low water content could delay the burst time and could elevate the initial burst temperature, but its burst was a single and more fierce failure, which could be ascribed to the effect of water within the concrete. With the increase in temperature, the water in the test specimen would be gradually transformed into steam and aggregate continuously to the internal part with a lower temperature, and the concrete developed a spalling burst when the steam pressure was greater than the concrete tensile strength. The test specimen with a high water content had contained sufficient water, and the large amount of water in the interior would make it impossible for the water to move from the exterior with a higher temperature towards the interior with a lower temperature; as a result, steam would be formed near the test specimen surface, and the exterior would first suffer from spalling when the steam pressure was excessively high. However, there was a low water content within the test specimen with a low water content, which could leave enough space for the internal migration of external water; the internal water had continuously aggregated, the steam pressure was continuously increased and no steam pressure-induced spalling was observed near the surface, since there was no water, but it fiercely burst when the internal steam pressure was large enough. This was consistent with the steam pressure theory. 

### 4.3. Effect of Size of the Fiber Concrete Test Specimens

**Figure 12:**
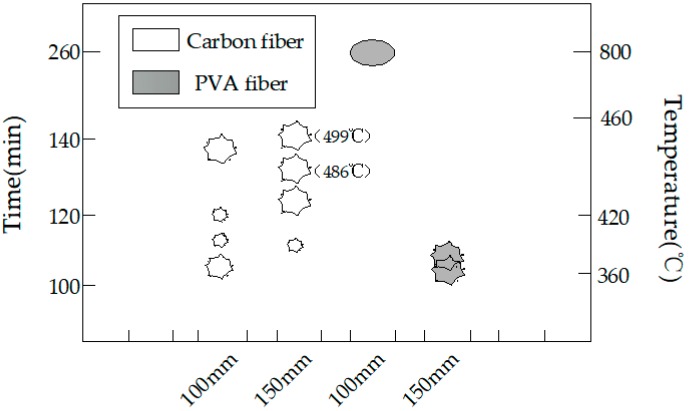
The burst statistics of concrete with different sizes.

**Figure 13:**
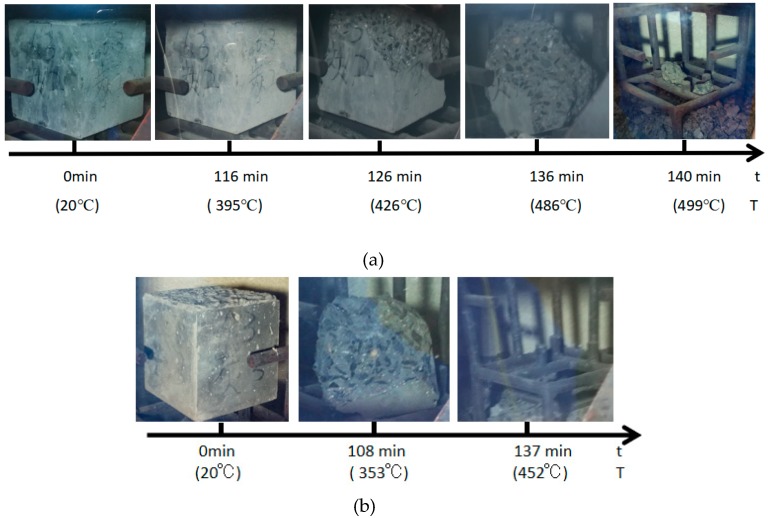
The explosion process of the carbon fiber specimens with different specimen sizes: (**a**) the 150-mm cube concrete specimen with carbon fiber and (**b**) the 100-mm cube concrete specimen with carbon.

The burst statistics are shown in Figure 12. All test specimens had burst apart from the PVA 100 mm test specimen. The burst process of the carbon fiber test specimen is presented in Figure 13. It was discovered in experiment that the carbon fiber 150 mm cube had a longer burst process, the lateral edges of the test specimen were subject to spalling at 116 min and the furnace temperature at this time was 395 °C; at 126 min, spalling could be observed at the edges and corners of the upper test specimen, along with bursts to various degrees on the surface, and the furnace temperature at this time was 426 °C; and at 136 min, all six planes of the test specimen suffered from spalling burst, while the remaining test specimen globally approximated a sphere, and the furnace temperature at this time was 486 °C. At 140 min, the test specimen had completely burst, and the furnace temperature at this time was 499 °C (Figure 13a). 

By contrast, the carbon fiber 100-mm test specimen under the same conditions had developed sparse bursts at 108 min, followed by a continuous burst until a complete burst at 137 min (Figure 13b). 

**Figure 14:**
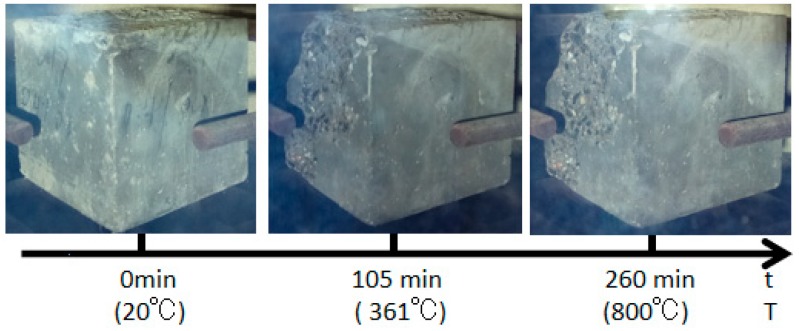
The explosion process of the 150-mm PVA specimens.

The PVA 100-mm test specimen did not burst, while the PVA 150-mm test specimen had spalling on the surface at 105 min, as presented in Figure 8b and Figure 14, and the furnace temperature at this time was 361 °C; afterwards, no secondary spalling phenomenon was observed. Thus, it could be figured out that, for the 150-mm test specimen, the addition of 0.4% PVA fiber could not completely suppress the burst, since size had a great influence on the burst of high-strength concrete. In the interior of a large size test specimen, the water escape and migration path became longer and the water in the outmost layer could escape into the air during the initial heating process, while water that was far away from the surface could only migrate to the interior with a lower temperature; in this way, a saturated water layer was gradually formed in the interior, which could restrict the release of internal pressure and thereby lead to bursting. When the internal-external distance became longer, the temperature gradient between the center and the furnace temperature was so large that water could not escape in a timely manner, leading to bursting [34]. 

### 4.4. Effect of Heating Rate

**Figure 15:**
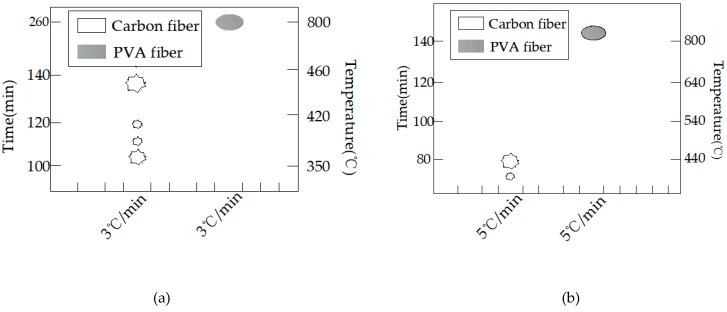
The burst statistics of concrete with different heating rates: (**a**) A heat rate of 3 °C/min and (**b**) a heat rate of 5 °C/min.

**Figure 16:**
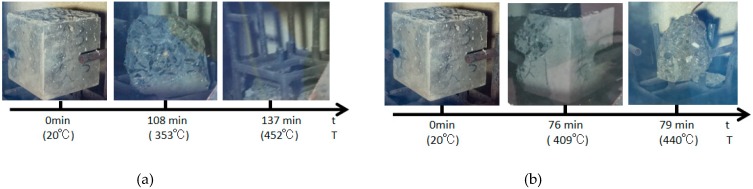
The explosion process of the carbon fiber specimens with different heat rates: (**a**) a heat rate of 3 °C/min and (**b**) a heat rate of 5 °C/min.

The burst statistics of the test specimens at two heating rates are displayed in Figure 15. The carbon fiber test specimen had burst, but the PVA test specimen did not burst. At the heating rate of 3 °C/min, the carbon fiber test specimen had developed two bursts; the first one occurred at 108 min at a furnace temperature of 353 °C, and the upper half of the test specimen had burst; the second one occurred at 137 min at a furnace temperature of 452 °C, which was the continuous burst in Figure 16a. At the heating rate of 5 °C min, the carbon fiber test specimen had also developed two bursts; the first one occurred at 76 min at a furnace temperature of 409 °C, and the vertical corner of the test specimen had burst; the second one occurred at 79 min at a furnace temperature of 440 °C (Figure 16b). 

**Figure 17:**
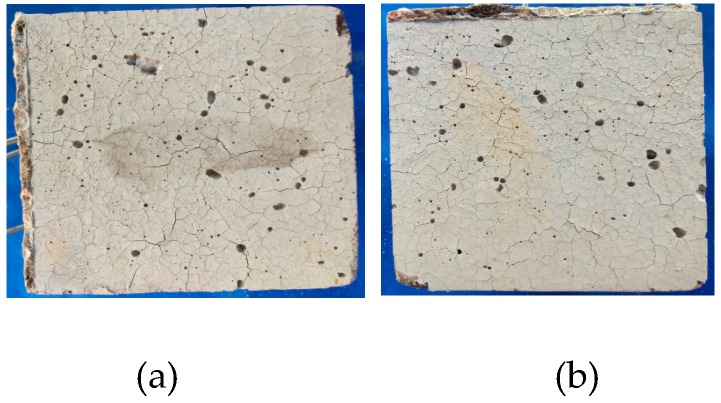
The final state of the PVA fiber specimens with different heat rates: (**a**) A heat rate of 3 °C/min and (**b**) a heat rate of 5 °C/min.

The PVA test specimen did not burst, but intense cracks could be observed on the surface, as presented in Figure 17.

The test results suggested that, compared with the heating rate of 3 °C/min, the heating rate of 5 °C/min could advance the burst time of the test specimen while shortening the burst duration, indicating that the steam pressure within the concrete would be increased at a faster rate in the presence of a higher heating rate and that the initial burst time would be advanced. However, the excessive heating rate would render a sudden increase in the thermal radiation on the concrete; as a result, the external heat could not be timely and effectively transferred into the interior due to the limited thermal conductance of the concrete and the fiber. In this way, there was a great difference between the internal and external temperatures along with a thermal expansion of the concrete to various degrees in different regions; after reaching a certain difference, the internal stress produced by thermal expansion was greater than the interaction between the concrete materials, finally giving rise to a burst [16]. 

## 5. SEM Samples

**Figure 18:**
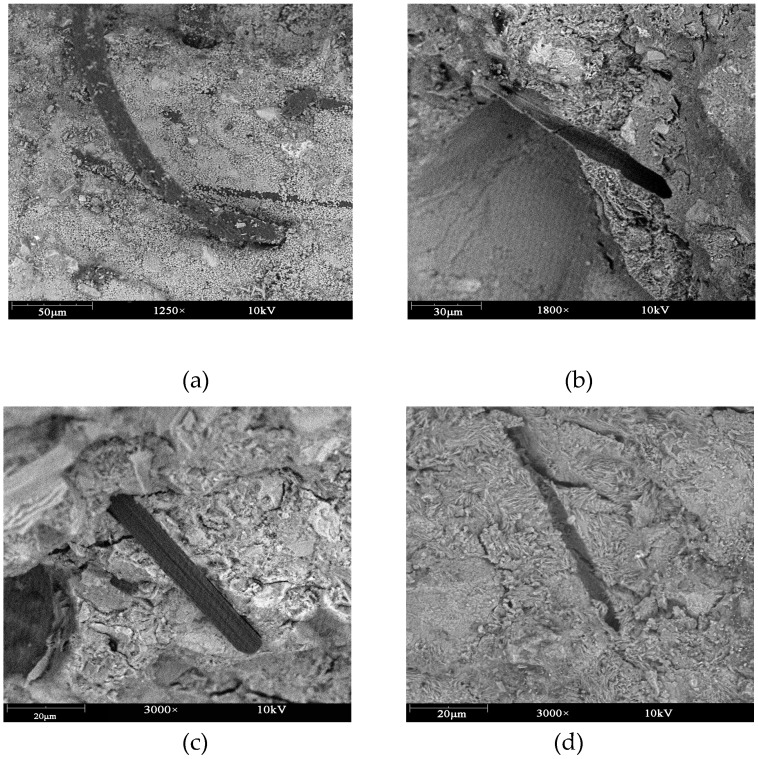
The final state of the PVA fiber specimens with different heat rates: (**a**) PVA at 25 °C; (**b**) PVA after 250 °C; (**c**) carbon fiber after 600 °C; and (**d**) carbon fiber after 800 °C.

As shown in Figure 18a,b, the PVA fibers have been gasified at 250 °C to form pore channels, while concrete without fibers will burst at about 426 °C. Therefore, the pore channels of PVA can release the vapor pressure to suppress the bursting. Carbon fibers still exist after 600 °C, as shown in Figure 18c,d. Therefore, the reason why carbon fibers inhibit bursting is due to their high thermal conductivity and good mechanical properties. Carbon fiber also forms carbon dioxide and pores after 800 °C, which may cause a decline of the mechanical properties of concrete.

## 6. Conclusions

From the observation and the records of the experiments, some useful results were obtained and are given below:

1. Fiber addition will improve the high-temperature burst behavior of the high-strength concrete, and the performance of PVA is greatly different from that of carbon fiber.
2. Carbon fiber can markedly improve the strength of the high-strength concrete but cannot suppress the incidence of bursting; instead, it can only change the sudden burst into a continuous weak burst. In this experiment, 0.2% carbon fiber is the optimal addition amount, but the addition of carbon fiber will reduce the burst temperature of the concrete, and the reduction is related to the amount of addition of carbon fiber in the high-strength concrete. Generally, the temperature will be 42–73 °C lower than that of the basic mix proportion.
3. PVA fiber has a superb burst-suppression property; its addition can enhance the flexural strength and splitting strength of the concrete, and it has little influence on the axial compressive strength but will reduce the compressive strength of the concrete. PVA fibers will develop vaporization under high temperatures, and holes will be formed in the concrete, which can effectively release the concrete vapor pressure and suppress the incidence of concrete burst. The influence of the mixed addition of fiber on the concrete is the same as that on the PVA test specimen.
4. The water content and heating rate have little influence on the burst of the PVA test specimen, but they will greatly affect the carbon fiber test specimen. A low water content will delay the initial burst time of the carbon fiber test specimen, but the burst will become fiercer. Similarly, a faster heating rate will advance the initial burst time of the carbon fiber concrete and accelerate the burst degree.
5. The size of the test specimen has a great influence on the burst. For the PVA concrete test specimen, the large size test specimen burst on the surface; as for the carbon fiber test specimen, the large size test specimen delayed the initial burst time, but the burst became fiercer.

## Figures and Tables

**Table 1:** The experimental design.

Materials	Quality (kg/m^3^)	Volume Fraction of Fibers		
		Carbon Fiber (PO)	PVA Fiber (PO)	Carbon + PVA Fiber (PO)
Cement	570	0 (12.8) C2 (13.41) C4 (14.36) C6 (16.87)	0 (12.8) P2 (13.32) P4 (14.66) P6 (17.01)	0 (12.8) P1C1 (14.56) P2C1 (16.93) P2C2 (16.99)
Silicone	60			
Fly ash	60			
mineral powder	80			
PO	12.8			
Water	144			
Fine aggregate	622			
Coarse aggregate	930			

Note: PO: Polycarboxylate superplasticizer.

**Table 2:** The properties of the fiber used in the experiments.

Properties	Carbon Fiber (C)	Polyvinyl Alcohol Fiber (PVA)
Specific mass (g/cm^3^)	1.98	1.3
Fiber length (mm)	6	9
Fiber diameter (μm)	7	12
Melting point (°C)	3500	230
Tensile strength (N/mm^2^)	4200	1200
Young modules (N/mm^2^)	230,000	21,000

**Table 3:** The precise test conditions.

Test Content	Type	Content	Moisture Content	Size	Heating Up System
Effect of fiber	C, PVA, C + PVA	0.2, 0.3, 0.4, 0.6	Normal	100 mm	3 °C/min
Effect of moisture content	C, PVA	0.4	Dry, Normal	100 mm	3 °C/min
Effect of heating up system	C, PVA	0.4	Normal	100 mm	3 °C/min 5 °C/min
Effect of size	C, PVA	0.4	Normal	100 mm/150 mm	3 °C/min

**Table 4:** Mechanics test value of concrete at room temperature.

	Comp (MPa)			Spli (MPa)			Flex (MPa)			Axial (MPa)		
content (%)	1	2	3	1	2	3	1	2	3	1	2	3
0	106.5	107.1	104.4	5.8	5.4	6.5	10.2	10.3	8.9	75	81.3	77.7
P2	100	95.4	86.6	6.0	6.2	7.1	12.2	13	11.7	87	87.5	80.5
P4	88	87.2	82.8	6.4	6.4	6.6	12	13.2	13.5	75	82.7	86.2
P6	86	89.6	88.4	5.9	6.3	6.9	12.2	12.0	13.2	90	84.7	74.3
C2	112	116.7	110.3	9.0	7.9	8.6	14.4	13.8	15.6	110.6	115.6	114.1
C4	98	107.7	112.3	7.7	7.9	7	14.0	14.6	13.6	96.3	106.9	102.8
C6	96.8	111.4	117	6.8	7.7	6.8	13.2	13.0	12.2	94	102.9	103.1
P1C1	101.6	97.4	89	6.7	7.4	6.9	14.6	13.6	14.9	78	86	88
P2C1	96	97.1	94.3	6.6	7.4	6.4	12.6	12.3	13.5	80	93	85
P2C2	84	88	95	7.3	7.5	6.8	12.0	12.8	12.7	94	86.7	82.1

Note: comp: Compression strength; Spli: Splitting strength; Flex: Flexural strength; Axial: Axial compression.

**Table 5:** The work ability of high-strength concretes.

Type	P0C0	P0C2	P0C4	P0C6	P2C0	P4C0	P6C0	P1C1	P2C1	P2C2
Slump (mm)	215	223	210	210	220	215	200	205	211	203
